# Vector Angular Continuity in the Fusion of Coseismic Deformations at Multiple Optical Correlation Scales

**DOI:** 10.3390/s23156677

**Published:** 2023-07-26

**Authors:** Rui Guo, Qiming Zeng, Shangzong Lu

**Affiliations:** Institute of Remote Sensing and Geographic Information System, School of Earth and Space Sciences, Peking University, Beijing 100871, China; grace@stu.pku.edu.cn (R.G.);

**Keywords:** deformation angle continuity, coseismic deformation, optical remote sensing, Maduo earthquake

## Abstract

As one of the common techniques for measuring coseismic deformations, optical image correlation techniques are capable of overcoming the drawbacks of inadequate coherence and phase blurring which can occur in radar interferometry, as well as the problem of low spatial resolution in radar pixel offset tracking. However, the scales of the correlation window in optical image correlation techniques typically influence the results; the conventional SAR POT method faces a fundamental trade-off between the accuracy of matching and the preservation of details in the correlation window size. This study regards coseismic deformation as a two-dimensional vector, and develops a new post-processing workflow called VACI-OIC to reduce the dependence of shift estimation on the size of the correlation window. This paper takes the coseismic deformations in both the east–west and north–south directions into account at the same time, treating them as vectors, while also considering the similarity of displacement between adjacent points on the surface. Herein, the angular continuity index of the coseismic deformation vector was proposed as a more reasonable constraint condition to fuse the deformation field results obtained by optical image correlation across different correlation window. Taking the earthquake of 2021 in Maduo, China, as the study area, the deformation with the highest spatial resolution in the violent surface rupture area was determined (which could not be provided by SAR data). Compared to the results of single-scale optical correlation, the presented results were more uniform (i.e., more consistent with published results). At the same time, the proposed index also detected the strip fracture zone of the earthquake with impressive clarity.

## 1. Introduction

As one of the severest forms of natural disaster, earthquakes seriously threaten the safety of human life and property. As such, coseismic deformation is an essential source of data for assisting the parameter estimation of surface deformation models during devastating earthquakes [1]. Satellite imagery is naturally suited for such measurements, because it regularly provides comprehensive and detailed images of the ground with high radiometric and geometric quality [2]. Interferometric Synthetic Aperture Radar (InSAR) is one of the most commonly used techniques to provide this data. However, when the coseismic deformation and gradient are large, InSAR faces some challenges, including severe phase decoherence and phase ambiguity. In many cases, these techniques do not offer detailed maps of near-field surface strain, which can be characterized by a complex network of surface ruptures and cracks within a fault zone of finite width. Therefore, InSAR and field measurements are not effective for estimating the total slip across a fault zone and the variability of slip along the fault [3]. Under these conditions, subpixel correlation methods based on image intensity information are more advantageous, such as optical image correlation (OIC) and pixel offset tracking (POT). Since the measurement accuracy of POT is primarily determined by the image’s pixel size, and the resolution of SAR images is significantly lower than that of optical images, OIC can generally determine the horizontal displacement more precisely than POT [1]. Furthermore, SAR systems often have longer repetition periods, resulting in more severe temporal de-correlation of image pairs, preventing POT from generating complete deformation fields in the near fracture zone. With an improved understanding of sampling theory, the correlation technique now provides local displacement measurements with precision up to a hundredth of a pixel. This is made possible by enhanced interpolation capabilities [4].

Co-registration of Optically Sensed Images and Correlation (COSI-Corr) allows for more accurate and robust measurement of the relative displacement between two images [5]. COSI-Corr has introduced a methodology for automatic and precise ortho-rectification and co-registration of satellite or aerial images. This procedure eliminates the need for external sources such as GPS-based ground control points (GCPs), and solely relies on topography and ancillary data provided with the observing platform. The generated set of ortho-images is then used for sub-pixel change detection. It has produced validated results in many earthquake studies [6,7,8,9,10,11]. In addition, the technology is also widely used in dune displacement, glacier displacement, and other fields [12].

Even so, COSI-Corr still has some problems which need to be solved. Firstly, general optical images with lower or medium spatial resolution are occasionally insufficient to measure fine tomographic displacements [13]. This can be dealt with via higher spatial resolution optical remote sensing imaging, which can expand the applicability of the technology and improve measurement accuracy. The second issue is related to the matching window size, which is an important algorithmic parameter of the correlation because it determines the measurement scale of the frequency correlation and directly affects the maximum displacement which can be measured [12]. The larger the correlation window, the lower the correlation uncertainty and bias [13], while in the opposite direction, smaller correlation windows close to the surface deformation produce more erroneous signals which manifest as noise [12]. In fact, this problem exists in most methods based on the pixel offset tracking principle. For example, Jiehua Cai et al. pointed out the dependence of offset estimation on correlation window size in the SAR POT technique [14]. However, there is limited research on this problem in optical data processing. Currently, the common methods for selecting the correlation scales are the traversal method or the empirical values reference method [11], but the former is less efficient, whereas the latter is influenced more by the characteristics of the test area.

One particularly notable case was the 2021 Maduo earthquake, to which four different correlation scale sizes were applied, where the variation among the maximum values approached a dozen meters. Even based on these four different outcomes, it was impossible to determine the most reliable technique. A simple average was also attempted for post-processing to seek results agreement, but the effect was not ideal. Furthermore, traditional post-processing divided the east–west and north–south directions without establishing a typical two-dimensional relationship. Therefore, considering the similarity of adjacent surface deformations, high-resolution optical remote sensing data were used to fuse different OIC deformation results for this earthquake, from which consistent results for multi-scale correlation shape changes could be obtained. The method’s reliability was demonstrated by comparing its results with those of Sentinel-1-based D-InSAR, POT, and other published studies.

## 2. Maduo Earthquake and Data Processing

### 2.1. Maduo Earthquake and Data

On 22 May 2021, a magnitude 7.4 earthquake occurred in Maduo County, Guoluo Tibetan Autonomous Prefecture, Qinghai Province, China. The earthquake took place about 70 km south of the East Kunlun Fault Zone on the northern boundary of the Bayankala block and was caused by an internal fault within the block. The Bayankara massif is extensively exposed to Triassic flysch construction. The strata are controlled by the regional tectonic movement, and the strata strike in the same direction as the regional tectonic line. The earthquake’s surface deformation was extensive, creating a complex coseismic deformation zone. The overall deformation showed a left-lateral strike-slip motion with a minor component of reverse faulting. The rupture mainly occurred along the Jiangcuo fault located in the southern part of the East Kunlun Fault Zone, with an overall trend of N105° E and a length of approximately 151 km [15]. Field investigations indicated that the earthquake caused several meters of surface displacement and triggered many fissures, intermittent springs, and seismic uplifts. The eastern and western parts of the fault experienced the most severe stretching and squeezing deformation. Finding out the distribution and characteristics of its coseismic surface rupture in time is of great significance for the identification of seismogenic structure, regional earthquake prevention, and disaster reduction [16]. The research area of this paper is mainly located on the south side of Cuergara Lake (a small lake on the south side of Eling Lake). According to the field seismic and geological investigation conducted by GAI Hailong et al., the tensional fractures in the study area are arranged in a right-order en echelon, with a general strike of 40°~60° and a width ranging from 10 cm to 3 m. The extrusion bulge is mainly distributed in the left-order oblique column, with a single strike of about 275°~280° and a height ranging from 0.3 m to 1.5 m, with a maximum of 1.6 m [16].

The optical remote sensing images used in this article are high spatial resolution data from the Planet satellite, which is a new type of remote sensing satellite with high-resolution, high-frequency, and global coverage capabilities [17]. This article conducted experiments using two PS Scene 3Band products, acquired on 8 May and 5 June, located in the western part of the Maduo earthquake rupture zone. The product has a positioning accuracy of less than 10 m RMSE, has been orthorectified using SRTM, and has a spatial resolution of 3.125 m. The common area of the two images, which was obtained after cropping, was selected as the study area, with an area of approximately 397 km^2^.

**Figure 1:**
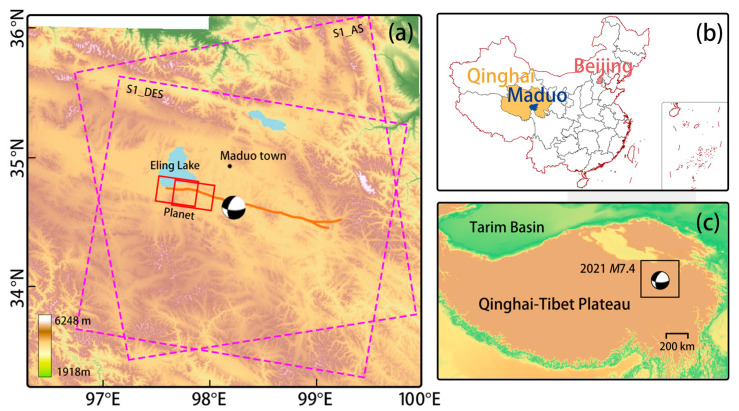
(**a**) Colour shadow relief map of the study area. Elevation information is based on ASTER GDEM 30 M. Optical data are represented by a solid red line, while SAR data are represented by a dashed purple line. The yellow-brown line represents the Maduo earthquake fault trace [18]. Beachball’s focal mechanism and epicenter location are based on USGS data, with the location of Maduo County shown as a black dot. (**b**) shows the relative position of the Maduo county in Qinghai Province in China. (**c**) shows the location of the study area on the Tibetan Plateau, and the black box in the figure is (**a**).

In the method validation section of this article, radar data were used to obtain coseismic deformation in the study area, serving as a reference and comparison for this article’s method. The radar data were collected on 20 May and 26 May, ascending- and descending Sentinel-1 satellite C-band data. The study area and data range are shown in Figure 1. In Figure 1a on the left, remote sensing data are marked. The larger purple box designates SAR data, and the flight direction is indicated above the box in the form of abbreviations, AS for ascending orbit and DS for descending orbit. Two smaller red boxes from left to right indicate the optical data before and after the earthquake.

### 2.2. D-InSAR and POT Processing

We used the GAMMA2020 software to process the collected Sentinel-1 acquisitions with the D-InSAR and POT techniques. Initially, the single and multi-look complex images (SLC) from the upward and downward Sentinel-1 Synthetic Aperture Radar (SAR) data were extracted for corresponding bursts in respective areas. Using the satellite orbital parameters, the Shuttle Radar Topography Mission (SRTM) digital elevation model (DEM) with a resolution of 90 m was converted to the radar coordinate system. The coarse and fine registrations of the interferometric pairs of ascending and descending SLC data were performed to improve azimuthal registration accuracy. By eliminating the terrain phase and the flat earth phase by DEM and SAR orbit parameters, respectively, the raw differential interferogram was obtained. To suppress phase noise and improve the signal-to-noise ratio (SNR), multi-looking processing was performed with azimuthal and range views 10 and 2, respectively. An adaptive filter was applied to the obtained interferograms to eliminate phase noise and reduce the difficulty of phase unwrapping. To improve the accuracy of phase unwrapping, the low-coherence areas were masked before performing two-dimensional phase unwrapping and converting the deformation phase to displacement values in the LOS direction. Finally, the obtained interferograms were transformed to the geographic coordinate system through geocoding to produce the coseismic interferogram and deformation field for ascending and descending SAR images.

**Figure 2:**
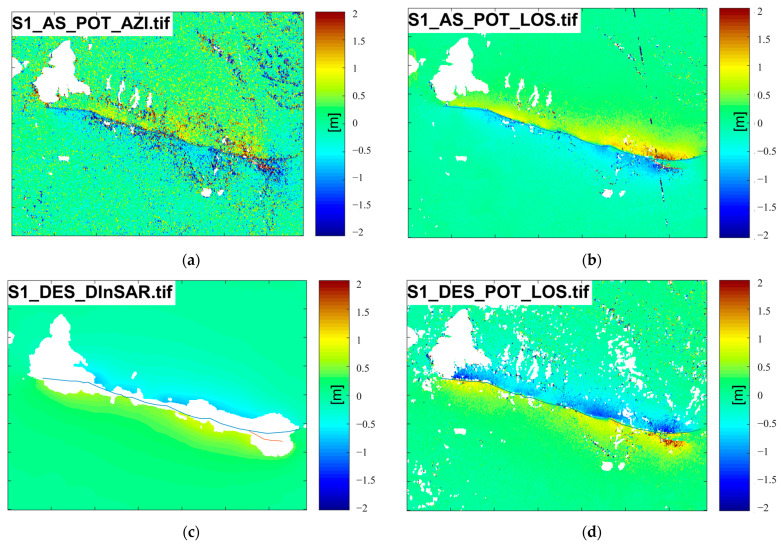
Deformation obtained by D-InSAR and POT methods using Sentinel-1 ascending and descending data, respectively, where the blue line is the marked rupture zone. (**a**) is the line-of-sight displacement obtained after POT processing of the ascending data; (**b**) is the LOS displacement obtained after POT processing of the ascending data; (**c**) is the result obtained after D-INSAR processing of the descending data; (**d**) is the LOS displacement obtained after POT processing of descending data.

Figure 2 depicts the coseismic displacement observations obtained from SAR data. To represent the corresponding SAR displacement observations, we use a combination of several abbreviated letters connected by underscore characters. For example, S1_AS_POT_AZI denotes the POT observation of ascending (AS) Sentine1-1 (S1) data along the AZI direction, whereas S1_DES_D-InSAR denotes the D-InSAR observation of descending S1 data. The trend and distribution of displacement observations are similar and consistent with the results in published papers [15,16,18,19,20,21,22,23,24,25,26,27].

Severe decoherence and sizable missing areas are noticeable in the InSAR results. The POT generates more noise, and the POT results from downgraded data show small missing areas in the epicenter region. This phenomenon occurs due to the extreme horizontal displacement changes in these regions.

According to data from the China Continental Tectonic Environment Monitoring Network, a permanent east–west displacement of up to 25 cm has been recorded at the Maduo station, located over 30 km away from the epicenter. Meanwhile, the InSAR also detected significant deformation, with the maximum relative Line of Sight (LOS) displacement of about 1.87 m and 2.32 m in ascending and descending directions, respectively [19]. Our measurements are consistent with published studies, so SAR measurements can be used as reference data for validation of subsequent optical imaging methods. A qualitative comparison showed that POT observation results were less accurate than D-InSAR observation results, and were proportional to pixel resolution. The results confirmed a crucial advantage of the POT method, which can provide more complete displacement field information than D-InSAR in areas where the latter method is infeasible. Then, using the method proposed by Liu et al. [28,29], the deformation was decomposed into the east–west and north–south directions to compare it with the method proposed in this paper, which is based on optical data.

### 2.3. Optical Image Correlation

While COSI-Corr has been designed to extract sub-pixel displacements between optical images, it is important to acknowledge that factors such as uncorrected topography, noise, and limited resolution can impact measurement results despite maximizing image utilization [30]. When calculating pixel offset through correlation analysis, there are two correlators available-frequency and statistical. Among the two, the frequency correlator, which is based on Fourier analysis, is more accurate than the statistical one. It should be prioritized when using optical images for offset tracking. However, the frequency correlator is highly sensitive to noise and is recommended only for optical images of good quality. On the other hand, the statistical correlator maximizes the absolute value of the correlation coefficient and is more robust. Although it is coarser than the frequency correlator, it is useful in correlating noisy optical images that have yielded unsatisfactory results using the frequency correlator. Additionally, the statistical correlator is also ideal for images with different contents, such as an optical image combined with a shaded digital elevation model (DEM).

The frequency correlation process involves two main steps. The first step requires an initial estimation of the pixelwise displacement between two patches. If the image is noisy or there is an expected large displacement, a large initial window size should be selected. In theory, the window size should be twice the expected displacement. However, it is recommended to use a larger ratio, such as a 128 or 256 initial window size with an expected displacement of 15 pixels. Once the initial displacement is estimated, a second and final correlation step is performed to retrieve the subpixel displacement. For the final window size, it is customary to choose the smallest size that provides an acceptable level of noise. The researchers suggest that a window size of 32 × 32 pixels is the minimum necessary, while a window size of either 32 × 32 or 64 × 64 is usually sufficient to yield good results with high-quality images [30]. This is because smaller window sizes increase the density of independent measurements.

**Figure 3:**
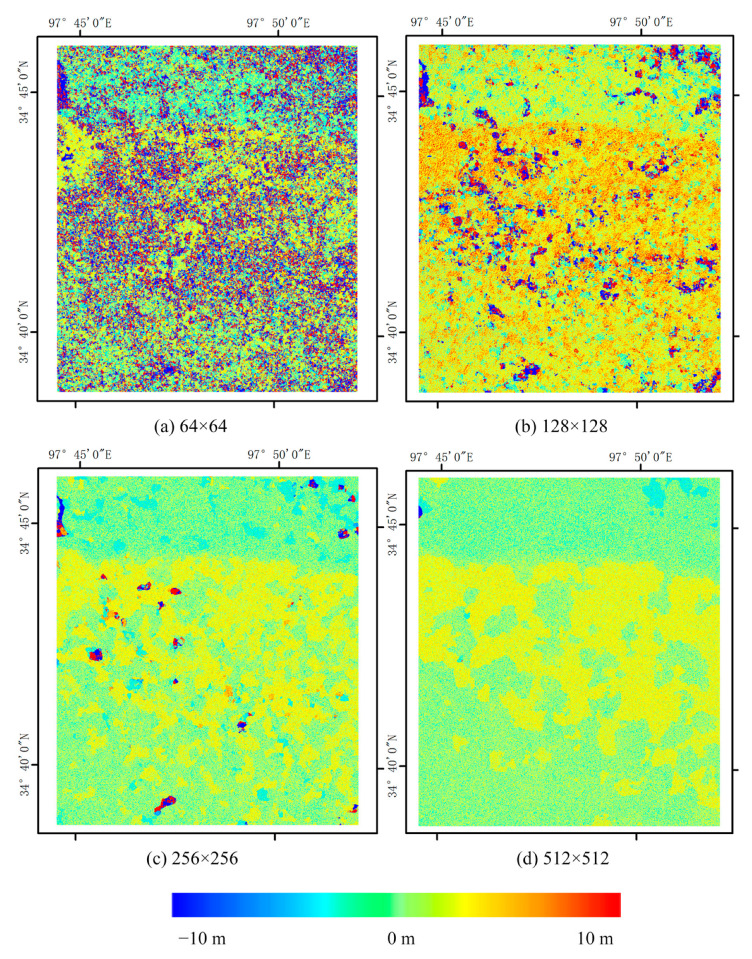
East–west (E–W) deformation for various correlation scales (**a**–**d**), where the eastward direction is positive. The number represents the size of the matching window used, and the spatial resolution is 6 m in all images.

**Figure 4:**
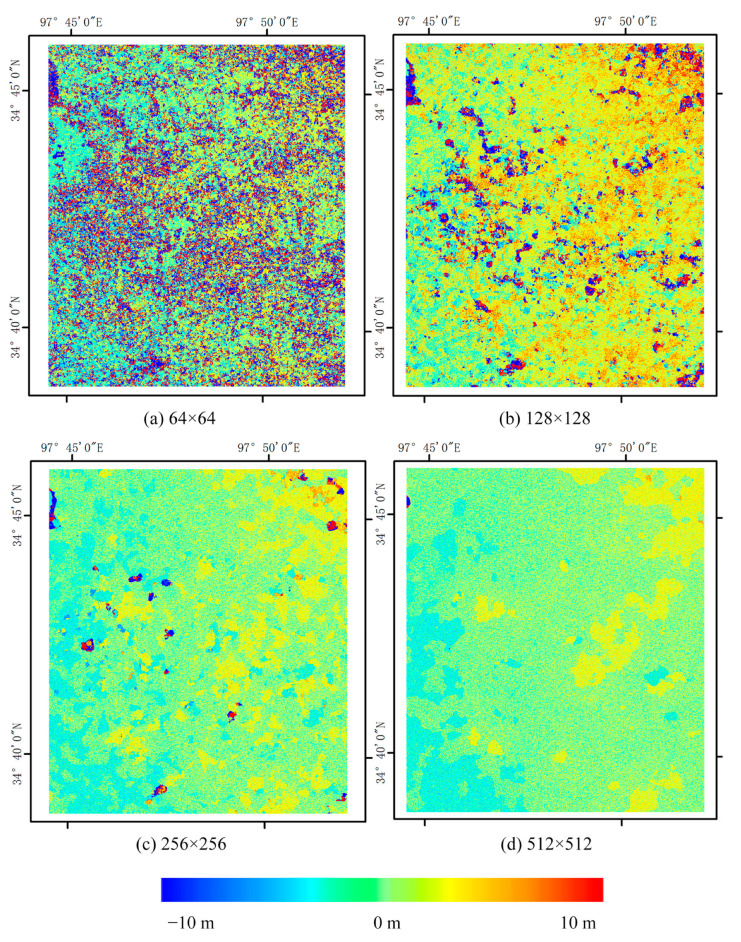
North–south (N–S) deformation for various correlation scales (**a**–**d**), where the northward direction is positive. The number represents the size of the matching window used, and the spatial resolution is 6 m in all images.

So in this paper, in order to explore the effect of different correlation windows on the results, with the same settings for the remaining parameters (step size of 2 pixels, number of iterations of 2, and signal-to-noise ratio threshold set to the default value of 0.9), correlation was conducted by COSI-Corr at scales of 64 × 64, 128 × 128, 256 × 256, and 512 × 512 pixels. The east–west and south–north displacements are obtained. Where north and east are positive, south and west are negative. The displacement results are shown in Figure 3 and Figure 4 as below, which demonstrate that the higher spatial resolution of optical data provides richer detailed information. Moreover, the variations in the coseismic displacement values obtained from the different correlation windows are more noticeable. The average displacement is positively correlated with correlation window size, and the noise significantly decreases under a larger window.

**Figure 5:**
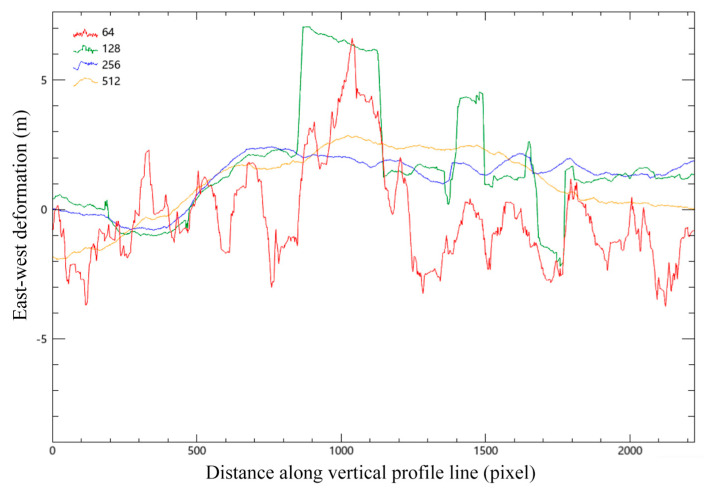
Vertical profile line analysis of the east–west deformation (around the 550th column of the abscissa was the location of the rupture zone); number in the upper left corner is the size of the relevant window, e.g., 64 × 64 pixels, they are represented in different colors.

A vertical section line is drawn in the middle of the image, and the change of coseismic deformation with the distance of the section line under each window is analyzed, as shown in Figure 5.

To select the appropriate fusion scale, profile analysis was carried out, which determined that the four windows detected faults in the same column. The 64 × 64 pixels window was similar to its 128 × 128 counterpart, wherein both exhibited more wavelet peaks. Analogously, the 256 × 256 and 512 × 512 pixels windows were also similar. Overall, across all window sizes, the performance was relatively flat. Nevertheless, determining which method was correct or incorrect was difficult, hence the attempt to fuse the results from a range of correlation scales to achieve greater uniformity. According to the above analysis, the subsequent steps selected the 128 × 128 and 256 × 256 pixels windows for interpolation fusion.

## 3. VACI Fusion Method

To address the issue of the correlation technology of optical images being affected by the size of the correlation window, this study employs a vector analysis method to fuse deformation results of varying scales during the correlation process. The study considers the spatial continuity of the deformation vector and applies weighted fusion to the results of differently sized correlation windows for optical images.

**Figure 6:**
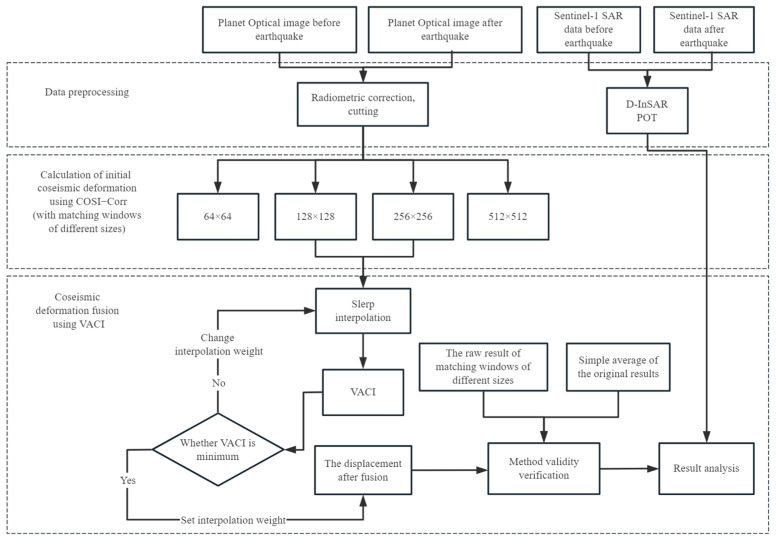
The technical flow chart of this article.

With the exception of fault rupture areas, the surface displacements of adjacent points are usually correlated in geophysical processes such as earthquakes, and ignoring this correlation limits the accuracy of determining displacement [18]. Taking this into consideration, the Vector Angular Continuity Index (VACI) of coseismic deformation was used to determine the pixel-by-pixel adaptive fusion weights, and then the optical cross-correlation offsets of different size correlation windows in the above process are fused together. The following Figure 6 is the technical flow chart of this article.

### 3.1. Coseismic Deformation Vector Angle Continuity Index

Displacement fields resulting from coseismic or inter-seismic events can be represented by a three-dimensional vector for any given ground point. In the local reference system, this vector may be expressed as its north, east, and vertical components. When generating a 2D displacement image, each pixel represents a single ground point displacement measurement. This is achieved by taking the scalar product of the real displacement and a vector (including north, east, and vertical components) that represents the projection of the measurement onto the local reference system [4]. The deformation components in the east–west and north–south directions were analyzed simultaneously in this paper. To do so, a two-dimensional vector $v=\left(x,y\right)$ was used to represent the horizontal displacement of each pixel point by designating the east–west and north–south directions as the x- and y-axes, respectively (eastward and northward being positive directions). The angle between the deformation vector and the eastward direction was defined as the horizontal coseismic deformation angle, which was calculated by the arc-cosine function.

If each pixel center is taken as the origin, with the east-west direction as the x-axis and the north–south direction as the y-axis, with the positive direction being eastward and northward, then the actual horizontal displacement of each pixel can be represented by a two-dimensional vector, denoted as $\vec{v_{1}}=\left(x_{1},y_{1}\right)$, where the subscript number represents the scale order. The cosine of the deformation angle and the deformation magnitude can be calculated. Generally, within a certain observation scale, in a certain area far away from the fault with uniform geological characteristics, the spatial distribution of coseismic deformation angles derived from optical images should exhibit a certain continuity. In the high spatial resolution optical images used in this article, there should be no significant jumps between adjacent pixels. Considering each pixel and its eight neighboring pixels, the displacement vectors can be represented by their coordinates.

To study the variations of the deformation angle between adjacent pixels, it is necessary to calculate the angle between two adjacent vectors. Two calculation methods are proposed: one is to calculate the angle between the two vectors directly using their dot product, i.e.,

(1) $$\cos\alpha =\frac{x_{1}\cdot x_{2}+y_{1}\cdot y_{2}}{\sqrt{x_{1}^{2}+y_{1}^{2}}\cdot \sqrt{x_{2}^{2}+y_{2}^{2}}}$$

The other is to first calculate the angle between each vector and a reference positive direction (horizontal east) separately, denoted as:

(2) $$\cos\theta_{1}=\frac{x_{1}}{\sqrt{{x_{1}}^{2}+{y_{1}}^{2}}}$$

(3) $$\cos\theta_{2}=\frac{x_{2}}{\sqrt{{x_{2}}^{2}+{y_{2}}^{2}}}$$

Then take their difference:

(4) $$\Delta \theta =\theta_{1}-\theta_{2}=\alpha$$

After standardizing all deformation vectors, the formulas for calculating the angles using the two methods are:

(5) $$\alpha_{1}=\arccos(x_{1}\cdot x_{2}+y_{1}\cdot y_{2})$$

(6) $$\alpha_{2}=arccos\left(x_{1}\right)-arccos(x_{2})$$

The Vector Angular Continuity Index (VACI) is defined as the average absolute difference in angle between the displacement vectors of a pixel and the eight surrounding pixels. This index describes the variation in displacement direction between a pixel and its neighboring pixels. The range of values for VACI should be between 0 and 1, and the unit is in radians. A higher VACI value indicates a larger difference in displacement direction between a pixel and its neighboring pixels, reflecting a more discontinuous and fragmented surface deformation in the neighborhood. A smaller VACI value indicates a smaller difference in displacement direction, reflecting a more continuous and consistent deformation direction in the neighborhood. Below are the formulas for calculating VACI using the two aforementioned methods:

(7) $$\mathrm{VAC}I_{1}=\left|\frac{\sum_{i=1}^{9}\alpha_{i}}{8}\right|=\left|\frac{\sum_{i=1}^{9}\arccos(x_{i}\cdot x_{5}+y_{i}\cdot y_{5})}{8}\right|$$

(8) $$\mathrm{VAC}I_{2}=\left|\frac{\sum_{i=1}^{9}\left(\arccos(x_{i})-\arccos(x_{5}\right))}{8}\right|=\left|\frac{\sum_{i=1}^{9}\arccos(x_{i})-9\arccos(x_{5})}{8}\right|$$

where *I* ranges from 1 to 9. Due to the lower time complexity, the first method of calculation is chosen.

**Figure 7:**
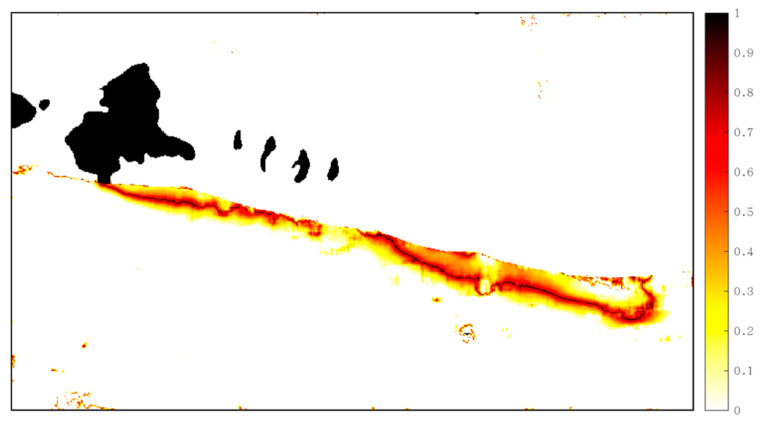
VACI index distribution of Sentinel-1′s POT results, in which the black line segment with a larger value is consistent with the rupture zone (except for lakes). The value of VACI in the image is dimensionless.

To test the reliability of VACI in detecting the angular properties of deformation vectors in the rupture and non-rupture zones, its distribution with respect to the deformation results obtained using POT from Sentinel-1 was calculated (Figure 7). The VACI value was clearly greater along the rupture zone, reaching approximately 0.8 radians, and lower in other, more removed, areas. It was demonstrated that aside from the area adjacent to the rupture zone, the horizontal deformation vector retained better angular continuity even when the majority of the surface exhibited definite coseismic displacement. VACI suppressed the obvious burrs in the original image and revealed an obvious rupture zone in the near east direction of the Jiangcuo fault (which extends eastward from Eling Lake and bifurcates in the southeast). This was consistent with previously published optical images and analytical results [25,27]. As a result of phase incoherency, a silhouette surrounding the rupture zone appeared.

### 3.2. Multi-Scale Coseismic Deformation Field Fusion

To comprehensively describe the deformation characteristics at different deformation-related scales, the deformation vectors obtained at different scales are merged. The common merging method is to simply average the east–west and north–south components separately. However, from the perspective of vector analysis, this merging process is actually an interpolation process for two vectors of different sizes and directions. If a simple linear interpolation is used, the endpoint of the computed vector will always fall on the path between the difference of the two vectors, and the size of the resulting vector will always be smaller than the original vector, which is obviously not consistent with the actual situation of coseismic deformation vectors. Therefore, the Spherical Linear Interpolation (SLERP) method commonly used in computer graphics to describe vector movement is considered, since it has long been used in computer animation to interpolate movements between two 3D orientations [31]. The weight which minimizes VACI was selected by testing a range of values (i.e., it was chosen based on the best angle direction continuity of the coseismic deformation vector).

(9) $$\vec{v(t)}=\frac{\sin(1-t)\alpha }{\sin\alpha }\vec{v_{1}}+\frac{\sin t\alpha }{\sin\alpha }\vec{v_{2}}$$

$\vec{v_{1}}$ and $\vec{v_{2}}$ are the original deformation vectors obtained at different correlation scales, and α is their angle. $\vec{v(t)}$ is the deformation vector obtained after interpolation. t is the weight, determined by VACI. The SLERP interpolation results are constrained by the angle continuity index VACI proposed above; the interpolated deformation vector VACI value is calculated and selected to be the minimum, that is, the weight with the best angle direction continuity of the coseismic deformation vector.

## 4. Results and Discussion

Various errors exist in the horizontal deformation field obtained from data processing, including decorrelation noise, orbital errors, and terrain shadow errors. For decorrelation noise, a signal-to-noise ratio (SNR) threshold of 0.9 is set in the deformation field, and a mask is used to remove regions with an SNR below the threshold. Since there are no large mountains or terrain undulations in the study area, terrain shadow errors are not considered. As optical image correlation methods theoretically cannot detect deformations greater than half the size of the correlation window, extremely large and small deformation values greater than half the window size are replaced with null values, and the missing values are then filled using inverse distance weighting, thereby removing decorrelation noise.

Orbital errors appear in the deformation results as a global intrinsic quantity and are often removed using a polynomial surface fitting model. Reference points are established in the non-deformation areas, and the desired parameters are solved using the least-squares principle. The simulated orbit error component of the whole image is subtracted from the original deformation field to obtain the true surface deformation field [9].

**Figure 8:**
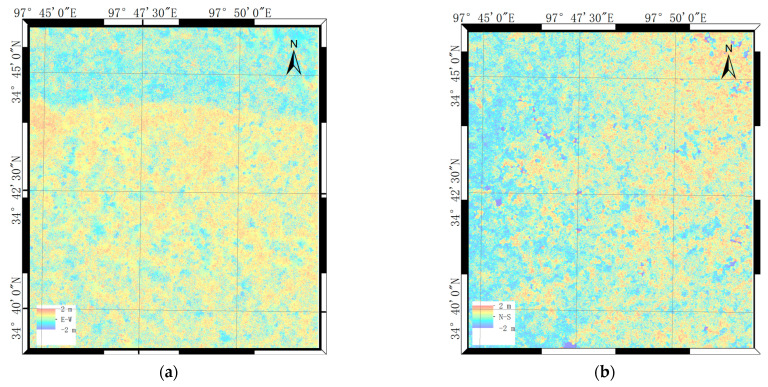
(**a**) East–west and (**b**) north–south deformations.

The final result obtained by using the VACI method introduced in Part Three after error processing is displayed in Figure 8. The strike-slips on the north and south sides of the epicentral rupture zone can be clearly discerned in Figure 8a.

### 4.1. Comparison with SAR Data

**Figure 9:**
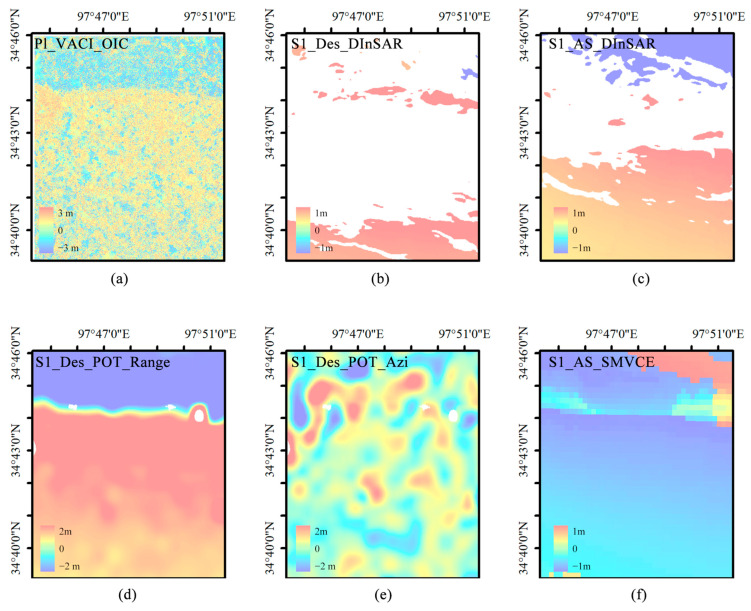
(**a**) The modulus of the proposed method’s results, whose positive and negative components are defined by the east–west component, and the east–west displacement component is regular and positive; (**b**,**c**) the LOS deformation of the D-InSAR method for Sentinel-1 data; (**d**,**e**) distance and LOS deformations of the Sentinel-1 orbit descent POT method; (**f**) the two-dimensional deformation, where the eastward direction is positive, calculated by the strain model and variance component estimation (SMVCE) method. Reprinted/adapted with permission from Ref. [28]. 2018, Jihong Liu.

In order to verify the accuracy of optical data fusion results, the VACI OIC method results were compared to those obtained via the traditional D-InSAR and SAR POT techniques for the southern part of Eling Lake and the western end of the rupture zone as shown in Figure 9. To sort the various sets of data, a combination of several abbreviated letters connected by underscore characters was used to represent the corresponding displacement observations (e.g., ‘S1_AS_POT_AZI’ denotes the POT observation of ascending Sentine1-1 data along the AZI direction, whereas ‘S1_DES_D-InSAR’ denotes the D-InSAR observation of descending S1 data).

Severe decoherence and sizable missing areas were noticeable in the InSAR results. Meanwhile, the POT generated more noise, and its results from downgraded data showed small areas missing from the region around the epicenter. This phenomenon was observed due to the extreme horizontal displacement changes which occurred in these regions. The modulus of the deformation vector was calculated to facilitate comparison. Deformation data from areas with poor coherence could be extracted using optical remote sensing images, and the resolution which resulted from this was much higher than that of POT. In remote sensing technology for obtaining coseismic deformation, optical image correlation has advantages such as high precision and immunity to the influence of decorrelation in the epicentral area. Moreover, POT was only able to obtain more intuitive results in the distance direction, while the Sentinel-1 orbit descent POT method results were very blurred and almost no deformation could be seen in them.

Among the methods surveyed, SAR POT is the one that best matches the results of this study and provides superior visual effects. Its spatial resolution, however, is limited, and it struggles to capture detailed features (although such limitations are not well demonstrated in this study). In contrast, the use of high-resolution optical imagery, illustrated in Figure 9a, enables higher-resolution estimates. This advantage is mainly due to the characteristics of optical data sources. In our processing, the original optical cross-correlation offset calculation results were post-processed under the constraints of geophysical significance. This approach involved optimizing the window size and resulted in a less noisy and more reliable estimate, which in turn significantly improved the signal-to-noise ratio of the original optical cross-correlation offset calculation results. When using optical images, in order to further analyze the effect of our proposed VACI fusion method on reducing the correlation window size, we also must compare the results with ordinary average fusion.

### 4.2. Accuracy Analysis

**Figure 10:**
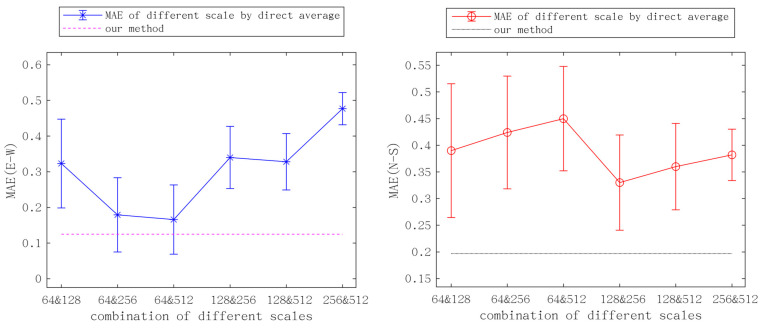
The mean absolute error (MAE) and standard deviation (STD), as calculated utilizing SAR POT as a reference. East–west and north–south are depicted in the left and right images, respectively. The plotted points denote the simple average of the different correlation scales from the ordinary OIC method, while the dotted line represents the proposed method.

In order to verify that the method proposed in this paper is much more effective than simply averaging the results of randomly selecting two sizes of correlation windows, we conducted pairwise averaging of the east–west and north–south results obtained by four sizes of correlation windows, taking SAR POT results as reference. Their mean absolute error (MAE) is calculated, and their standard deviation (STD) is plotted in the form of an error bar to compare with our method, as shown in Figure 10.

This method significantly reduces MAE. It is worth noting that compared with the fusion results of the same correlation scale, the trends of north–south deformation and east–west deformation are not the same, and the mean MAE of north-south results is higher. For the result using a 64 × 64 pixels window, adding a larger window improves the accuracy of its east–west results, but reduces the accuracy of its north–south results. For the result using a 512 × 512 pixels window, adding smaller window results improves the accuracy of its east–west and north–south results (except for the north–south shift fused with a 64 × 64 pixels window). This observation shows that the correlation scale and deformation direction are not uniform, which confirms the influence of the choice of correlation scale on the results. Therefore, in order to improve the performance of point target analysis in non-uniform moving regions, it is crucial to accurately evaluate the similarity of two candidate pixels, especially when using small-scale correlation windows.

The accuracy of the proposed method is better than that of any combination of Windows. The constraint based on displacement vector angular continuity avoids the above problems, and the adaptive weight can better adjust the balance of larger or smaller correlation windows. Additionally, the proposed method also significantly improved the peak signal to noise ratio (PSNR) and reduced the root mean square error (RMSE) as shown in Table 1. The accuracy of the results of the east–west deformation is significantly improved, with a 99% reduction in STD and a 74% reduction in MAE. The STD of north–south deformation is reduced by 95%, and MAE is reduced by 56%. Despite the fact that this method is based on the fusion of two scales, the results were shown to be superior to any single scale’s results, and the east–west and south–north directions show the greatest improvements.

## 5. Conclusions

When using the optical correlation offset tracking technique to detect coseismic deformation, the spatial sampling and measurement accuracy depend greatly on the local image correlation. The variability of the phase shift on the scale of the sliding window provides an estimation of the measurement’s consistency [32]. Although large correlation windows enhance measurement accuracy, they compromise resolution and blur small-scale motion details [14]. Therefore, achieving a balance between different window sizes is crucial for accurately evaluating pixel similarity. The size of the correlation window directly affects the reliability and accuracy of the results. In order to reduce the influence of different correlation window sizes on the results, this paper attempts to merge the deformation window results of large and small scales, with the aim of obtaining a deformation vector field that is superior to any single correlation scale.

In this work, the conventional correlation of surface displacements between adjacent points in the non-fault rupture region was considered. This offered an innovative approach, taking the resonance deformation vector as the research object and including the angular continuity index of the deformation vector (VACI). This index was implemented as a constraint to fuse the deformation results of different correlation scales obtained by OIC to obtain a vector field better than any relevant individual scale. Taking the 2021 Maduo earthquake as the subject of our proposal, high-resolution optical images allowed the generation of two-dimensional deformation fields and vector maps of the study area, from which the rupture zone was successfully detected via VACI.

The effectiveness of the method we proposed here was demonstrated by comparison with the traditional single correlation scale and the simple average of two-scale combinations, which showed that the accuracy of the east–west deformation results was clearly improved. Additionally, in contrast to POT technology, our processing process was able to provide a two-dimensional deformation field with higher spatial resolution and local detail information. When compared to D-InSAR, our method demonstrated superior effectiveness with respect to information from areas with large deformations and severe decoherence near the rupture zone. However, the deformation field obtained in this study only accounted for a small portion of the area affected by the Maduo earthquake deformation field, due to the limited range of available Planet images. Consequently, the VACI was defined within the context of only eight neighborhoods. Therefore, where this parameter can be improved is to use more neighborhood pixels or to adaptively determine the number of surrounding pixels used based on the distance from the epicenter, such that more surrounding pixels are considered, which may be more appropriate for large correlation scales.

## Figures and Tables

**Table 1:** A comparison of the RMSE and PSNR values of the proposed method versus a single correlation scale.

		64 × 64	128 × 128	256 × 256	512 × 512	Proposed Method
**RMSE**	**E-W**	18.79	15.48	8.45	3.70	0.98
	**N-S**	18.79	15.50	8.77	3.43	0.86
**PSNR**	**E-W**	22.65	24.34	29.59	36.78	48.27
	**N-S**	22.65	24.33	29.27	37.44	49.46

## Data Availability

Publicly available datasets were analyzed in this study. These data can be found here: https://www.planet.com (accessed on 7 June 2022). COSI-Corr is freely available from the California Institute of Technology’s Tectonics Observatory: http://www.tectonics.caltech.edu (accessed on 7 June 2022).
